# Home-Based Measurements of Nocturnal Cardiac Parasympathetic Activity in Athletes during Return to Sport after Sport-Related Concussion

**DOI:** 10.3390/s23094190

**Published:** 2023-04-22

**Authors:** Anne Carina Delling, Rasmus Jakobsmeyer, Jessica Coenen, Nele Christiansen, Claus Reinsberger

**Affiliations:** 1 Institute of Sports Medicine, Department of Exercise and Health, Paderborn University, 33098 Paderborn, Germany; 2 Division of Epilepsy and Clinical Neurophysiology, Department of Neurology, Brigham and Women’s Hospital, Boston, MA 02115, USA

**Keywords:** sport-related concussion, return to sport, autonomic nervous system, HRV, RMSSD, sleep, home-based

## Abstract

Sport-related concussions (SRC) are characterized by impaired autonomic control. Heart rate variability (HRV) offers easily obtainable diagnostic approaches to SRC-associated dysautonomia, but studies investigating HRV during sleep, a crucial time for post-traumatic cerebral regeneration, are relatively sparse. The aim of this study was to assess nocturnal HRV in athletes during their return to sports (RTS) after SRC in their home environment using wireless wrist sensors (E4, Empatica, Milan, Italy) and to explore possible relations with clinical concussion-associated sleep symptoms. Eighteen SRC athletes wore a wrist sensor obtaining photoplethysmographic data at night during RTS as well as one night after full clinical recovery post RTS (>3 weeks). Nocturnal heart rate and parasympathetic activity of HRV (RMSSD) were calculated and compared using the Mann–Whitney U Test to values of eighteen; matched by sex, age, sport, and expertise, control athletes underwent the identical protocol. During RTS, nocturnal RMSSD of SRC athletes (Mdn = 77.74 ms) showed a trend compared to controls (Mdn = 95.68 ms, *p* = 0.021, r = −0.382, *p* adjusted using false discovery rate = 0.126) and positively correlated to “drowsiness” (r = 0.523, *p* = 0.023, *p* adjusted = 0.046). Post RTS, no differences in RMSSD between groups were detected. The presented findings in nocturnal cardiac parasympathetic activity during nights of RTS in SRC athletes might be a result of concussion, although its relation to recovery still needs to be elucidated. Utilization of wireless sensors and wearable technologies in home-based settings offer a possibility to obtain helpful objective data in the management of SRC.

## 1. Introduction

Sport-related concussions (SRC) are common brain injuries classified as the mildest form of a mild traumatic brain injury (mTBI) [1]. They are caused by biomechanical forces transmitted to the head and provoking diffuse axonal damage, resulting in functional rather than structural disturbances [1]. Impaired autonomic control is frequently reported and observed following SRC and may last beyond clinical recovery, even when athletes adhere to the recommended protocol for stepwise return to sports (RTS) [2,3].

The parameters of heart rate variability (HRV) can easily be obtained to measure and monitor dysautonomia during SRC recovery. HRV offers valid indicators of (cardiac) parasympathetic activity (i.e., the root mean square of successive differences between normal RR intervals (RMSSD), the high frequency band (HF)) of the autonomic nervous system (ANS) [4]. Nocturnal HRV is suggested to be a sensitive indicator of homeostasis and was therefore applied in monitoring physiological recovery in athletes [5,6].

Resting HRV data during SRC recovery (i.e., with respect to exercise challenges) have frequently been examined [2], but to the best of our knowledge, recordings during sleep, one of the most important phases for cerebral regeneration [7], have not yet been investigated in acute SRC recovery. Wireless wrist sensors offer a convenient approach to continuously monitor autonomic activity (i.e., HRV) during sleep. They can be used in a home-based environment [8], overcoming the ecological limitations (e.g., expenditure, laboratory setting) of polysomnography (PSG), which is the clinical gold standard for sleep assessment. Sleep problems are one of the most commonly reported symptoms of SRC [9], whereas the clinical presentation of SRC symptoms is individual and might be diverse [1]. Clinical sleep symptoms may arise from SRC-induced disturbances within brain networks (e.g., central autonomic network) affecting sleep–wake regulation [10,11]. They are associated with more SRC-related symptoms in general and longer recovery periods [12,13].

The aim of this study was to explore nocturnal cardiac parasympathetic activity in SRC athletes during and post RTS in their home environment using a wireless wrist sensor. It was hypothesized that nocturnal cardiac parasympathetic activity (RMSSD) is reduced in athletes during RTS following SRC. The relation between nocturnal parasympathetic activity and reported sleep-associated concussion symptoms was subsequently investigated.

## 2. Materials and Methods

This study received an ethics approval from the local medical council (Westphalia Medical Board; approval number: 2019-147-f-S). It was conducted between April 2019 and December 2021 in accordance with the Declaration of Helsinki. Participants provided informed consent prior to their participation in this study.

### 2.1. Participants

In this study, 18 athletes after SRC and 18 healthy, matched controls participated. Subject characteristics are presented in Table 1. Demographics did not differ between the groups. Identification of SRC athletes was conducted through reports of (local) sports clubs, active screening of the news, as well as screening of patients attending the sports neurology clinic at the Institute of Sports Medicine at Paderborn University. In addition, 18 control athletes matched by sex, age, height, weight, sport, and expertise underwent the same protocol. Exclusion criteria for both groups included cardiovascular, neurological, or skin diseases prior to SRC, mental or physical disability, diabetes mellitus, and pregnancy. Control athletes were excluded if they had a concussion within the last year.

Evaluation of medical history included history of attention-deficit/hyperactivity disorder (ADHD), learning disability, migraine/headaches, as well as concussion history (number of previous concussions). Primary sport, position, and expertise level (elite, recreational) were documented. Concussion symptoms and symptom severity scores were retrieved from the symptom checklist of the “Sport Concussion Assessment Tool 5” (SCAT5) [1]. SCAT5 was applied once during the clinical neurological assessment or upon inclusion into the study. Symptom items “trouble falling asleep,” “drowsiness,” and “fatigue or low energy” were attributed to the sleep cluster [14,15,16].

Completion of the RTS protocol took on average 37 (±44) days. Three SRC athletes were included in this study after 26, 38, and 89 days after injury, because they were still suffering from clinical symptoms, requiring 124 ±40 days to finish RTS.

### 2.2. Study Protocol and Devices

SRC athletes received a wireless, wrist-worn multisensor (Empatica^®^ E4, Milan, Italy) [17]. The sensor recorded blood volume pulse (BVP) at a sampling rate of 64 Hz using photoplethysmography (PPG), from which heart rate (HR) and parameters of HRV (RMSSD) can be calculated. Previous research has shown that during rest and night measurements, the sensor exhibited valid PPG data in comparison to electrocardiography, resulting in consistent performance in both devices in over 85% of analyzed 24 h-data [18]. To control for movement, 3D-Accelerometry (32 Hz) was simultaneously recorded by the device. SRC athletes were instructed on how to wear and operate the sensor and test the device. They specifically were introduced to setting markers in the evening and in the morning after awakening, producing identifiable timestamps in the raw data. The sensor was worn on the non-dominant wrist during all nights of individual RTS. Full recovery was defined as completion of the last stage of the RTS protocol with medical clearance to return to normal competitive game play [1]. SRC athletes were asked to conduct one additional nocturnal recording three weeks after completion of RTS. Control athletes were instructed similarly to SRC patients and wore the sensor for the same number of nights except for the additional night after RTS.

### 2.3. Data Analysis

Nocturnal BVP data were processed to analyze HRV indices. Raw data was cut using a custom built script (Python, v. 3.9.12) to remove data not attributed to sleep. Night onset was defined as the first 10 min after the evening marker without any movement [19], which was visually determined using accelerometry data. Approximate wake-up time was defined using the morning marker. If the cut BVP data resulted in a data length less than four hours, the measurement was excluded from further analysis. This was the case for one (0.38%) of the control and one (0.30%) of the SRC athletes. The BVP segments were additionally preprocessed using Kubios HRV Premium (v. 3.5.0, Biosignal Analysis and Medical Imaging Group, Kuopio, Finland). The pulse acceptance threshold was set to 50%. Ectopic beats were removed by the automatic artifact correction algorithm and replaced by interpolated adjacent interbeat-intervals (IBI) in Kubios HRV Premium. The automatic noise detection (default setting: medium) of Kubios HRV Premium was applied to mark distorted IBI detections (e.g., caused by movement) as “noise segments” [20]. These segments were visually checked by one investigator (ACD), and the length was adjusted, if necessary. Noise segments were excluded from the analysis. Data containing more than 25% noise segments (effective data length < 75%) and data surpassing 10% of corrected IBI were excluded from further analysis.

Heart rate (HR) and root mean square of successive IBI differences (RMSSD) were analyzed within the final processed nocturnal BVP segments. Both variables have been demonstrated to be validly analyzed in data collected over a longer period of time [21]. RMSSD reflects cardiac parasympathetic activity [22] and was obtained from the whole nocturnal segment to reduce variation between different sleep stages [23]. To reduce day-to-day variation, the mean over all nocturnal recordings was calculated for each SRC and control athlete.

Normal distribution was assessed using the Shapiro–Wilk test. Between group comparisons (concussed vs. controls) for demographics, medical history, HR, and RMSSD were conducted using the Mann–Whitney U test. Effect sizes were calculated using Pearson’s r. To assess the extent of nocturnal variation of HR and RMSSD, the coefficient of variation (CV) was calculated for each athlete. Between-group analysis for CV was based on the Mann–Whitney U test. The coefficient of determination (R^2^) was calculated per group (last RMSSD versus mean RMSSD) to analyze how well the last data (i.e., post RTS) fits the linear model of the mean. A higher R^2^ indicates that the model (during RTS) better explains the variation in the dependent variable (post RTS). Mean was defined as “during RTS” for SRC and “mean over all nights except the last” for controls. Post was defined as “post RTS” for SRC and “last night” for controls. The study was conducted to explore the relation between sleep-associated concussion symptoms and nocturnal cardiac parasympathetic activity. The level of significance was defined as *p* ≤ 0.05. Due to the exploratory character of the study, uncorrected *p*-values as well as adjusted *p*-values (using the false discovery rate) were presented. All data were analyzed using SPSS (v.28, IBM Corporation, Armonk, NY, USA). Concussion symptoms and nocturnal cardiac parasympathetic activity were displayed as Median (Mdn) with standard deviation (±).

## 3. Results

### 3.1. Subjects and Concussion Symptoms

There were no group differences for age, weight, height, BMI, or number of previous concussions (Table 1). SRC athletes reported a greater number of symptoms (Mdn = 7; U = 25.500, z = −4.364, *p* < 0.001, r = −0.727, *p* adj. = < 0.01) and greater symptom severity (Mdn = 16; U = 13.000, z = −4.756, *p* < 0.001, r = −0.792, *p* adj. = < 0.01) on SCAT5 than controls (symptoms: Mdn = 1, severity: Mdn = 1). SRC athletes reported a significantly higher value in the sleep associated symptom “fatigue or low energy” (Mdn = 2; U = 58.000, z = −3.499, *p* < 0.001, r = −0.583, *p* adj. = < 0.01), while data showed a trend towards higher “drowsiness” (Mdn = 0) in comparison to controls (bothMdn = 0) after correcting for multiple testing (U = 104.500, z = −2.317, *p* = 0.025, r = −0.386, *p* adj. = 0.055) (Table 2). Six originally recruited participants were a priori excluded due to ectopic beats (*n* = 1), insufficient data quality (*n* = 1), not wearing the sensor accurately (*n* = 3), and drop out for personal reasons (*n* = 1).

### 3.2. Nocturnal Recordings

The individual course and duration of RTS resulted in a large variety of recorded nights per athlete. Due to time point of inclusion, technical issues, sickness not related to SRC and occasional non-adherence to the protocol by subjects, not all nights of the individual RTS were recorded. During RTS, the 18 SRC athletes produced 331 nocturnal measurements. Two hundred ninety-two of these nights (88%) were suitable for further analysis, resulting in a mean of 16 (±17) nights per athlete (range: 2–49). The first measurement was performed on average 13 (±21) days after SRC with a range from 1 to 90 days. Post RTS recording was conducted 28 (±20) days after RTS and on average 65 (±46) days after the injury. Control athletes engaged in 263 nocturnal recordings, from which 217 nights (83%) were included in the analysis (12 ±6 per athlete; range: 4–28). The discrepancy of nights between the groups was statistically not significant (U = 156.500, z = −0.175, *p* = 0.869). Data on individual RMSSD and HR data for each night during RTS, as well as individual CV for RMSSD and HR within each night, are presented in the Supplementary Materials (Tables S1–S3). The overall data quality was similar in both groups. Effective data length was 94.28% (±2.28%) in SRC and 94.71% (±3.48%) in controls, while the average interpolation rates of IBI were 0.96% (±0.35%) and 0.86% (±0.42%), respectively.

### 3.3. Nocturnal Heart Rate and Cardiac Parasympathetic Activity

**Figure 1:**
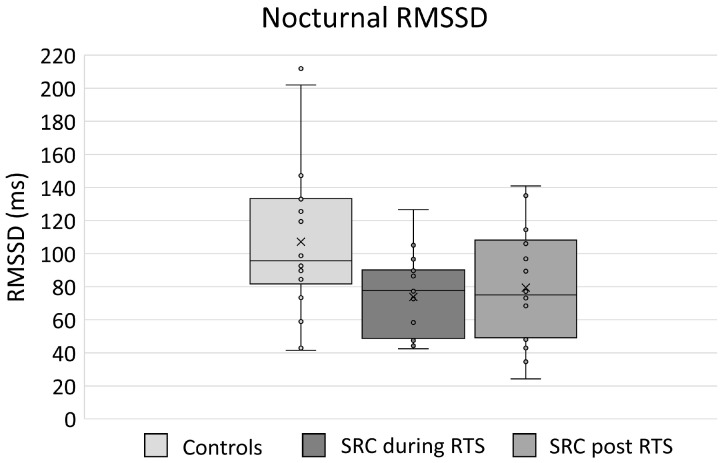
Nocturnal RMSSD for control and SRC athletes, during and post return to sport (RTS).

The results for nocturnal cardiac parasympathetic activity for SRC and control athletes are displayed in Table 3 and Figure 1. Nocturnal RMSSD (Mdn = 77.74 ms, *p* = 0.021, r = −0.382, *p* adj. = 0.126) during RTS was lower in SRC athletes compared to controls (Mdn = 95.68 ms) but not statistically significant after correcting for multiple comparisons. There was no difference in nocturnal HR between SRC (Mdn = 56 bpm) and controls (Mdn = 55 bpm, *p* = 0.515, *p* adj. = 0.567). CVs of HR (SRC: Mdn = 6%, Controls: Mdn = 5%, *p* = 0.491, *p* adj. = 0.567) and RMSSD (SRC: Mdn = 18%, Controls: Mdn = 13%, *p* = 0.199, *p* adj. = 0.398) did not differ between the groups during RTS. Post RTS, neither nocturnal RMSSD (SRC: Mdn = 75.12 ms, Controls: Mdn = 95.68 ms, *p* = 0.085, r = −0.290, *p* adj. = 0.255) nor nocturnal HR (SRC: Mdn = 55 bpm, Controls: Mdn = 55 bpm, *p* = 0.567, *p* adj. = 0.567) were different between SRC and control athletes. 

**Figure 2:**
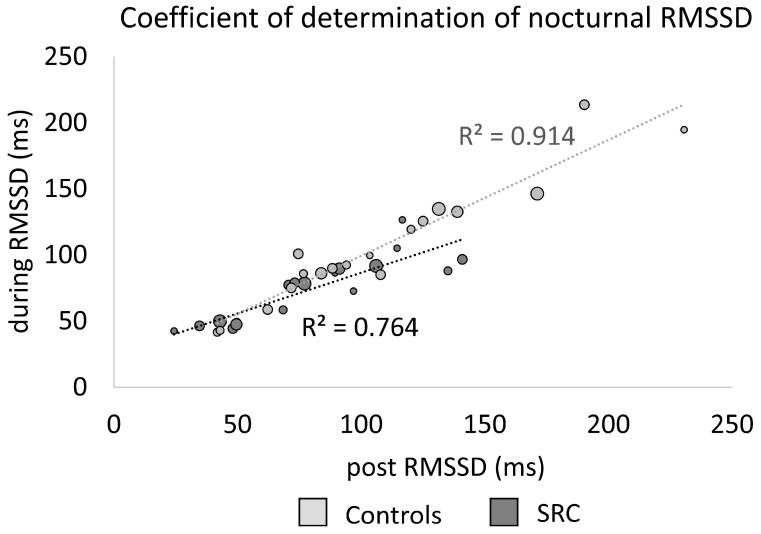
Coefficient of determination (R^2^) of the nocturnal RMSSD for SRC athletes (during RMSSD = mean during return to sport (RTS), post RMSSD = post RTS) and controls (during RMSSD = mean over all measurements except the last measurement, post RMSSD = last measurement). Larger dot sizes represent a higher number of measurements.

The coefficient of determination of nocturnal RMSSD during RTS and RMSSD post RTS (R^2^ = 0.764) for the SRC as well as mean RMSSD and last RMSSD (R^2^ = 0.914) for controls are visualized in Figure 2.

### 3.4. Correlations of Sleep-Associated Symptoms and Nocturnal Cardiac Parasympathetic Activity

While “fatigue or low energy” was reported significantly more often in SRC than in control athletes, there was only a trend towards significance in the symptom “drowsiness.” Analysis showed a positive correlation between nocturnal RMSSD during RTS and “drowsiness” (r = 0.532, *p* = 0.023, *p* adj. = 0.046), while there was no correlation between RMSSD during RTS and “fatigue or low energy” (r = 0.257, *p* = 0.304, *p* adj. = 0.304).

## 4. Discussion

This study aimed to explore nocturnal cardiac parasympathetic activity during and post RTS in athletes, following SRC and healthy, matched, control athletes in a home-based setting using a wireless wrist sensor. Differences in nocturnal cardiac parasympathetic activity during RTS lost statistical significance after correcting for multiple comparisons between SRC and control athletes. Post RTS (>3 weeks after RTS), no difference was detected. No group differences were found for nocturnal HR, whether during or post RTS.

Decreased cardiac parasympathetic activity following SRC was shown in studies investigating HRV with respect to physically challenging tasks, even in an asymptomatic state post SRC [24]. Heterogeneous and even conflicting results have been reported when focusing on isolated resting conditions post SRC [3,24,25]. One of the sparse studies investigating long-term HRV (24 h) examined concussed youth athletes (mainly females) and reported a decreased cardiac parasympathetic activity (the percentage of adjacent NN intervals differing from each other by more than 50 ms (pNN50), RMSSD, and HF) in the first 30–40 days post injury. An increase of parasympathetic activity was shown until day 75 post concussion, which might be indicative of compensatory processes, while no significant differences to the control group were found [26]. The results of the presented study also indicate no differences for RMSSD post RTS (65 ±46 days after SRC) between SRC and control athletes, although the coefficient of determination for RMSSD during and post RTS within the SRC athletes (R^2^ = 0.764) did not display the same degree of dependency as the controls (R^2^ = 0.914). This could potentially indicate incomplete recovery of cardiac parasympathetic recovery post RTS. Previous research hypothesized that physiological disturbances may persist even past resolution of clinical symptoms [27]. Hutchison et al. (2017) reported parasympathetic inhibition using short-term resting measurements within the first week after SRC in college athletes but also after symptom resolution and one week after RTS (RTS: 35 ±38 days post injury) [3]. Continuous monitoring beyond the RTS protocol, for example, comparing athletes with a long vs. short RTS and/or results of baseline testing, could provide a more precise insight into physiological recovery and potential dysautonomia following SRC in the future.

Nocturnal RMSSD correlated positively with “drowsiness” in the investigated SRC athletes during RTS. Since drowsiness was reported as mild (severity of 1 and 2, except for one athlete reporting 6), the clinical relevance of this finding remains unclear. “Trouble falling asleep,” a symptom of insomnia, was not regularly reported in the SRC group, indicating that this prominently described sleep problem after mTBI [28] might not have been problematic in this (high level) athlete cohort. A prolonged recovery and more SRC-related symptoms in general are commonly associated with subjective sleep symptoms reported within the acute stage following SRC [12,13], underpinning the importance of researching the link between dysautonomia and sleep disorders following SRC [14]. However, concussion symptoms were not collected again after inclusion into the study, so it remains unclear how symptom resolution developed individually.

This longitudinal investigation of nocturnal parasympathetic activity using a wireless wrist sensor in high-level athletes under home-based conditions indicated an applicable, non-invasive, and possibly even long-term approach to obtain objective physiological data in the home environment. As sleep represents a highly standardized measurement condition of the ANS, nocturnal HRV recordings are proposed to possess an enhanced reliability and accuracy [5]. More than 83% of the obtained recordings were of sufficient quality for further analysis. Data were mainly excluded due to interrupted measurements because of battery, data storage, or technical problems. The high compliance underlines the practicability and implementation of wearable sensors for monitoring sleep in an athletic population, while good feasibility and acceptability in studies using this sensor in clinical patient groups [29,30] have been reported.

There are several limitations that need to be considered when interpreting these explorative results. The time from the injury to recruitment was quite diverse and ranged from 1–90 days post SRC. Individual RTS ranged from 7–170 days, while the post RTS measurement ranged from 30–191 days, making this group quite heterogeneous in this regard. Since adult SRC athletes were mainly recruited from local elite sports clubs of contact and collision sports (soccer, basketball, American football) results may not be representative of the general athletic population. Missed documentation of training load and specific procedures during RTS possibly resulted in confounders that may also impact nocturnal parasympathetic parameters like physical deconditioning [31]. Nocturnal data were estimated on timestamps and identified according to lack of movement, resulting in an approximation of time in bed not equal to sleep. Mean parasympathetic activity over the whole night was determined, which might not display possible nocturnal dynamics or sleep cycles of HRV during sleep. Calculating a mean over all nights during the RTS was performed to reduce the day-to-day variation in ANS activity but prevented presentation of individual trends of cardiac parasympathetic activity during the individualized RTS.

Due to the exploratory character of this study, corrected as well as uncorrected *p*-values were reported. Although the group difference in RMSSD did not hold significance after adjusting for multiple comparisons, reported moderate effect sizes may encourage further investigation of the observed trend towards a reduced parasympathetic activity during sleep in athletes following SRC. Methodological shortcomings such as the small sample size and the approximation of sleep need to be considered. Individual neurophysiology (i.e., high intra- and intervariability in the ANS, larger levels of nocturnal parasympathetic activity in highly trained athletes) and the heterogeneity of the injury indicate additional confounders as well as relevant approaches, which need to be further explored in future studies. Moreover, the examination of individual dynamics of nocturnal ANS activity during and post RTS to better understand the mechanism induced by SRC and its possible association with (sleep-related) concussion symptoms should be considered in prospective studies.

## 5. Conclusions

Reduced nocturnal cardiac parasympathetic activity during RTS, but not post RTS, was observed in adult SRC athletes in comparison to control athletes but lost statistical significance after correcting for multiple comparisons. Parasympathetic activity correlated positively with the clinical symptom “drowsiness” in SRC athletes. As sleep indicates a fundamental regeneration period for the brain and provides valid measurement conditions avoiding influences of external factors on ANS activity [32], monitoring these restorative processes may aid management of SRC in the future.

## Figures and Tables

**Table 1:** Subject characteristics.

	Concussed (*n* = 18)	Controls (*n* = 18)	*p*
Sex	m = 15, f = 3	m = 15, f = 3	
Age (yrs.)	23 (±5)	23 (±5)	0.919
Height (m)	1.84 (±0.10)	1.84 (±0.10)	0.994
Weight (kg)	81 (±13)	81 (±14)	0.981
BMI (kg/m^2^)	24 (±2)	24 (±2)	0.833
Previous concussions	1 (0, 4)	1 (0, 4)	0.552
Sport	Soccer (8)	Soccer (7)	
	Basketball (3)	Basketball (4)	
	Handball (2)	Handball (1)	
	Am. Football (2)	Am. Football (4)	
	Ice Hockey (2)	Ice Hockey (1)	
	Modern Pentathlon (1)	Modern Pentathlon (1)	

Data are presented as group mean ± SD (min, max). Abbreviations: BMI, body mass index; m, male; f, female; Mann-Whitney U test.

**Table 2:** SRC symptoms (SCAT5) with significant differences between SRC and control athletes (Median ± SD).

	Concussed (*n* = 18)	Controls (*n* = 18)	*p* *	*p* (adj.)
Number of symptoms	7.00 (± 4.88)	0.50 (±4.20)	<0.001	<0.01
Symptom severity	15.50 (±15.54)	0.50 (±6.04)	<0.001	<0.01
Fatigue or low energy	2.00 (±1.77)	0.00 (±0.70)	<0.001	<0.01
Drowsiness	0.00 (±1.55)	0.00 (±0.51)	0.025	0.055
Headache	1.50 (±1.68)	0.00 (±0.00)	<0.001	<0.01
“Pressure in head”	2.00 (±1.41)	0.00 (±0.24)	<0.001	<0.01
Neck pain	1.00 (±1.42)	0.00 (±0.38)	0.002	0.006
Dizziness	0.00 (±0.86)	0.00 (±0.47)	0.036	0.072
Balance problems	0.00 (±1.04)	0.00 (±0.00)	0.045	0.083
Feeling slowed down	0.50 (±1.52)	0.00 (±0.24)	0.003	0.008
“Don’t feel right”	1.50 (±1.71)	0.00 (±0.24)	<0.001	<0.01
Difficulty concentrating	2.00 (±1.57)	0.00 (±0.24)	<0.001	<0.01
Difficulty remembering	0.50 (±1.26)	0.00 (±0.51)	0.011	0.026

* Mann-Whitney U test, *p* (adj.) = adjusted with false discovery rate.

**Table 3:** Results of nocturnal ANS activity (Median ± SD and 95% Confidence Interval [CI]).

	SRC During RTS	SRC Post RTS	Controls	Group Differences (*p*)		Group Differences (*p* adj.)	
				During RTS	Post RTS	During RTS	Post RTS
HR (bpm)	56 ± 6 [CI: 51–57]	55 ± 7 [CI: 50–57]	55 ± 8 [CI: 48–56]	0.515	0.567	0.567	0.567
RMSSD (ms)	77.74 ± 24.25 [CI: 61.78–85.90]	75.12 ± 34.41 [CI: 62.29–96.51]	95.68 ± 46.86 [CI: 83.87–130.48]	0.021 *	0.085	0.126	0.255
CV HR (in %)	5.50 ± 4.55	/	5.00 ± 2.55	0.491	/	0.567	/
CV RMSSD (in %)	17.50 ± 6.33	/	13.00 ± 7.63	0.199	/	0.398	/

HR: heart rate; RMSSD: root mean square of successive differences; RTS: return to sport; CV: coefficient of variation; * *p* < 0.05 (Mann-Whitney U test), *p* (adj.) = adjusted with false discovery rate.

## Data Availability

The data presented in this study are not available due to possible identification of subjects.

## Supplementary Materials

The following supporting information can be downloaded at: https://www.mdpi.com/article/10.3390/s23094190/s1, Table S1: RMSSD per night and coefficient of variability (CV) for each SCR and control athlete, Table S2: HR per night and coefficient of variability (CV) for each SCR and control athlete, Table S3: Individual variability of RMSSD and HR (CV) in the (RTS) nights for each SCR and control athlete.
