# Supplementary material for: Home-Based Measurements of Nocturnal Cardiac Parasympathetic Activity in Athletes during Return to Sport after Sport-Related Concussion
Source: Sensors (Basel). 2023 Apr 22;23(9):4190. doi: 10.3390/s23094190 (PMC10181314; doi:10.3390/s23094190)
Supplement: Supplementary file 1 [file sensors-23-04190-s001.zip › sensors-2225151-supplementary.pdf]

Supplementary Material

Table S1. RMSSD per night and coefficient of variability (CV) for each concussed and control athlete

	**Concussed**																	
**Subject ID**	3	5	9	10	11	13	14	17	18	19	20	21	22	24	27	29	30	31
**Nights**	**RMSSD (ms)**																	
**1**	70.76	49.32	48.11	51.81	105.63	114.97	97.68	38.63	94.39	102.26	85.51	37.72	51.49	86.26	116.52	83.01	52.92	43.65
**2**	74.61	40.86	36.97	74.19	113.95	107.17	84.77	47.38	96.76	102.47	89.79	45.46	52.98	103.93	102.05	79.14	51.43	61.51
**3**	57.92	36.91		102.63	93.32	109.65	89.93	53.43	79.38	98.55	83.77	46.74	44.21	76.64	154.44	75.65	50.55	64.85
**4**	87.59	42.41		81.82	92.34	94.24	76.21	35.97	90.78	73.11	92.73	47.53	49.45	66.15	133.36	82.75	45.65	64.51
**5**		46.60		86.82	111.57	74.78	83.84	45.06	84.34	77.55	60.30	52.65	51.08	76.85		85.72	44.77	57.75
**6**		51.89		78.73	113.88	92.80		61.57	80.71	84.71	68.26	38.54	60.24	108.17		92.34		58.00
**7**		43.76		108.85		66.22		31.98	78.40	108.36	99.72	44.88	55.42	103.27		75.38		
**8**		45.92		81.61		65.41		41.59	81.93	28.85	78.69	38.82	61.91	104.54		81.97		
**9**		50.69		52.59		71.22		50.68	90.34	88.45	103.09	42.42	56.82	83.81		78.51		
**10**		38.04		31.57		82.90		51.25	74.76	107.42	93.60	46.63	52.98	71.11		45.51		
**11**		47.89		83.66				56.16	85.50	113.58	85.34	38.93	49.92	76.75		80.59		
**12**		32.96		47.07				42.02	100.93	110.51	83.68	32.97	64.45	29.23		87.29		
**13**		52.93		83.45					88.47	129.77	74.61	52.10	56.36	115.56		76.62		
**14**		39.98		107.74					98.14	127.21	82.61	56.43	43.48	125.51		80.95		
**15**				86.36					93.70		65.89	50.44	58.03	92.52				
**16**									86.31		69.19	53.20	53.91	102.60				
**17**									110.19		66.37	51.25	75.08	80.62				
**18**									99.89		75.32	44.13	74.07	89.33				
**19**											74.75	47.90	51.25	66.03				
**20**											54.27	55.26	48.02	102.80				
**21**											64.26	56.14	39.99	89.24				

**22**											86.99	52.21	58.16	139.44				
**23**											64.94	61.23	59.80	121.33				
**24**											72.64	41.63	40.53	86.59				
**25**											68.41	38.52	37.37	117.03				
**26**											97.47	40.21	34.56	58.65				
**27**											80.07	60.23	60.55	78.97				
**28**											70.26	43.48	53.05	97.82				
**29**											78.34	57.13	53.01	108.43				
**30**											73.08	39.76	43.47	113.26				
**31**											86.64	46.75	40.53	93.63				
**32**											77.85	45.66	49.70	70.31				
**33**											72.44	60.09	34.69	110.33				
**34**												58.07	41.68	67.64				
**35**												58.13	38.99					
**36**												58.06	43.54					
**37**												52.55	45.37					
**38**												51.74	44.03					
**39**												38.69	43.89					
**40**												47.12	47.68					
**41**												50.31	49.64					
**42**												42.67	37.75					
**43**												26.90	45.56					
**44**												38.70	58.79					
**45**												50.75	44.31					
**46**												52.10	39.91					
**47**												40.99	50.03					
**48**													49.18					
**49**													40.59					

CV RMSSD between nights in %	17	14	19	29	10	21	9	19	10	27	15	25	18	24	18	14	7	13
	**Controls**																	
**Subject ID**	3.1	5.1	9.1	10.1	11.1	13.1	14.1	17.1	18.1	12.1	20.1	21.1	22.1	24.1	27.1	29.1	30.1	31.1
**Nights**	**RMSSD (ms)**																	
**1**	109.04	87.98	114.25	70.44	99.85	104.52	85.06	91.27	103.21	48.72	142.34	73.05	37.45	122.33	187.41	131.28	84.36	44.35
**2**	95.17	82.46	112.81	98.90	119.48	119.42	74.81	82.67	150.14	54.36	140.34	75.46	45.82	153.54	192.25	235.70	106.93	47.21
**3**	94.64	108.84	114.70	85.50	125.68	98.03	64.90	81.53	213.26	71.56	128.62	95.49	41.29	133.60	241.55	211.87	92.08	46.67
**4**	103.46	92.91	115.64	82.39	127.26	110.67	81.41	91.96	192.22	55.17	139.56	92.61	41.79	125.50	221.86	200.10	88.70	36.53
**5**		89.40	129.31	88.07	84.25	83.75	68.00	78.81	182.80	46.80	156.45	83.02		123.21	198.00	230.63	76.95	39.97
**6**		94.07	112.30	85.58	151.35	110.39	71.03	68.60	168.30	52.22	151.41	126.05		113.96	209.86		65.67	42.97
**7**			136.41	60.96	148.30	94.79	64.20	92.20	122.38	56.66	151.89	52.96		55.67	220.32		76.65	
**8**			120.17	70.86	123.44	98.31	80.97	71.26	83.91	49.23	152.91	112.91		129.26	221.08			
**9**				109.47	128.69	88.96	80.09	94.56	119.87	69.41	121.03	96.45		120.34	240.96			
**10**				103.15	148.14	104.04	83.14	86.39	108.60	59.12	151.27	85.16		157.21	229.58			
**11**				53.30	126.80	89.26	71.75	95.40	217.88	80.89	142.59	103.01		117.18	224.69			
**12**				100.74	123.05	106.89	55.17	86.31	68.61	62.69	142.05	81.84		179.53	196.99			
**13**				109.42	125.02			107.90	54.93	57.72	162.04			72.43	191.06			
**14**				85.96					94.86	57.63	153.84			226.02	190.30			
**15**				95.81					111.98	62.18	164.54			117.46				
**16**				84.36					129.32		170.87			145.02				
**17**				101.78					138.87		141.08			159.00				
**18**				63.30							165.44			178.56				
**19**				83.85							133.32			128.74				
**20**											135.83			131.44				
**21**											138.19							
**22**											157.75							
**23**											139.84							
**24**											127.96							
**25**											160.51							

**26**											152.67							
**27**											130.23							
**28**											141.94							
**CV RMSSD between nights in %**	7	10	7	19	15	13	13	12	36	16	9	21	8	27	9	21	16	10

Table S2. HR per night and coefficient of variability (CV) for each concussed and control athlete

	**Concussed**																	
**Subject ID**	3	5	9	10	11	13	14	17	18	19	20	21	22	24	27	29	30	31
**Nights**	**HR (bpm)**																	
**1**	49	54	54	66	44	44	39	57	54	48	62	54	55	57	44	41	64	65
**2**	48	58	54	59	47	46	42	55	52	50	62	52	56	55	47	42	63	57
**3**	49	60		53	47	46	42	53	56	49	60	51	58	60	44	44	66	58
**4**	46	57		55	49	46	45	59	56	56	58	51	59	61	43	44	67	57
**5**		55		53	45	55	45	57	58	57	63	49	60	60		44	68	57
**6**		54		56	46	50		53	58	53	68	54	59	56		42		55
**7**		64		52		55		60	60	48	60	51	59	54		49		
**8**		55		57		56		60	59	71	61	52	56	56		48		
**9**		56		63		56		57	60	50	58	54	58	58		49		
**10**		57		69		53		59	62	49	62	53	55	59		63		
**11**		56		59				57	61	45	59	54	59	60		48		
**12**		63		66				64	56	48	59	56	53	77		46		
**13**		53		55					59	47	63	50	55	54		48		
**14**		60		53					55	46	62	49	62	53		49		

**15**				56					58		65	53	56	56				
**16**									58		62	52	56	56				
**17**									53		65	52	54	55				
**18**									55		60	54	52	58				
**19**											64	54	55	58				
**20**											68	52	58	54				
**21**											66	51	63	58				
**22**											62	50	55	53				
**23**											64	49	54	56				
**24**											63	53	62	59				
**25**											66	54	64	54				
**26**											62	52	73	62				
**27**											60	48	55	59				
**28**											64	52	57	59				
**29**											62	49	56	59				
**30**											63	53	59	56				
**31**											59	54	63	59				
**32**											58	55	59	64				
**33**											60	53	60	58				
**34**												53	59	65				
**35**												52	61					
**36**												54	58					
**37**												54	58					
**38**												54	62					
**39**												58	60					
**40**												56	59					
**41**												54	58					
**42**												56	60					
**43**												62	58					

**44**												55	55					
**45**												52	59					
**46**												51	63					
**47**												54	56					
**48**													58					
**49**													60					
**CV HR between nights in %**	3	6	1	9	4	10	5	5	5	13	3	20	6	7	4	12	3	6
	**Controls**																	
**Subject ID**	3.1	5.1	9.1	10.1	11.1	13.1	14.1	17.1	18.1	12.1	20.1	21.1	22.1	24.1	27.1	29.1	30.1	31.1
**Nights**	**HR (bpm)**																	
**1**	41	53	38	59	57	54	51	59	52	59	57	60	64	69	36	50	40	51
**2**	45	53	38	54	52	53	52	60	49	59	57	61	62	58	39	44	41	52
**3**	48	49	37	57	59	58	56	61	49	54	56	57	61	58	37	47	38	52
**4**	42	49	42	55	56	58	49	59	46	60	58	55	60	61	40	48	39	59
**5**		49	38	58	74	59	56	60	49	60	56	59		62	36	44	39	55
**6**		49	37	55	55	58	55	64	50	59	58	51		66	37		40	53
**7**			42	61	54	55	56	59	51	58	58	66		81	36		40	
**8**			39	59	51	58	50	61	53	64	55	55		62	37			
**9**				54	50	56	53	60	54	55	60	55		63	39			
**10**				52	52	53	52	60	54	61	58	56		56	40			
**11**				63	49	56	53	57	46	52	55	56		61	38			
**12**				51	52	55	57	58	56	55	53	59		55	40			
**13**				54	50			57	58	58	55			75	35			
**14**				56					55	59	55			57	35			
**15**				55					53	56	57			64				
**16**				58					52		58			57				
**17**				52					50		62			62				
**18**				58							59			56				

**19**				56							61			60				
**20**											61			60				
**21**											57							
**22**											56							
**23**											54							
**24**											59							
**25**											56							
**26**											56							
**27**											62							
**28**											54							
**29**																		
**CV HR between nights in %**	7	4	5	5	12	4	5	3	6	5	4	7	3	11	5	6	2	5

Table S3. Individual variability of RMSSD and HR (CV) in the (RTS) nights for each concussed and control athlete

	**Concussed**																	
**Subject ID**	3	5	9	10	11	13	14	17	18	19	20	21	22	24	27	29	30	31
**Nights**	**CV RMSSD (in %)**																	
**1**	22	18	28	34	12	21	17	39	15	22	28	32	25	25	25	26	34	25
**2**	30	25	30	22	16	24	17	33	18	32	22	36	29	30	23	24	29	17
**3**	22	23		27	14	27	16	35	21	40	24	28	32	27	20	23	35	22
**4**	24	16		21	19	22	20	33	23	45	19	26	32	33	22	26	33	21
**5**		19		20	16	41	26	27	22	49	26	33	38	45		23	23	34
**6**		28		27	13	38		23	20	29	32	22	39	38		22		22
**7**		26		22		43		28	24	34	19	28	35	19		23		
**8**		16		22		37		27	18	32	24	27	26	31		20		
**9**		22		42		45		24	23	27	20	39	29	40		26		
**10**		24		48		43		25	28	24	29	37	24	30		34		
**11**		19		22				25	24	17	20	36	25	42		26		
**12**		32		36				33	17	24	26	34	27	45		21		
**13**		20		24					20	29	23	29	28	31		27		
**14**		23		22					19	23	28	26	32	21		21		
**15**				23					26		26	41	30	20				
**16**									21		24	33	30	29				
**17**									16		29	28	25	35				
**18**									23		19	33	29	38				
**19**											25	35	27	25				
**20**											28	36	29	27				
**21**											28	33	39	21				
**22**											20	28	25	27				
**23**											18	28	31	32				

**24**											23	33	32	45				
**25**											32	20	28	31				
**26**											25	31	56	25				
**27**											22	28	31	27				
**28**											33	36	25	31				
**29**											27	28	23	46				
**30**											20	35	33	19				
**31**											19	38	32	41				
**32**											22	39	28	54				
**33**											23	38	28	41				
**34**												41	28	40				
**35**												28	31					
**36**												46	35					
**37**												44	27					
**38**												34	30					
**39**												34	29					
**40**												36	28					
**41**												46	27					
**42**												39	28					
**43**												33	29					
**44**												29	26					
**45**												21	27					
**46**												28	39					
**47**												31	35					
**48**													33					
**49**													32					
**Mean CV RMSSD in %**	25	22	29	28	15	34	19	29	21	31	24	33	30	33	23	24	31	23

	Controls																	
**Subject ID**	3.1	5.1	9.1	10.1	11.1	13.1	14.1	17.1	18.1	12.1	20.1	21.1	22.1	24.1	27.1	29.1	30.1	31.1
**Nights**	**CV RMSSD (in %)**																	
**1**	16	22	14	31	25	30	28	19	41	21	21	29	25	57	12	44	26	16
**2**	20	21	12	22	28	28	26	18	49	25	19	20	27	13	14	17	30	18
**3**	15	17	14	24	31	39	27	19	58	23	21	17	24	19	15	47	27	21
**4**	16	21	23	20	30	38	27	16	35	33	16	15	22	33	12	54	26	25
**5**		19	14	25	68	45	33	18	62	25	15	20		37	12	13	21	14
**6**		17	17	15	33	38	36	17	72	23	21	21		44	15		17	22
**7**			15	38	25	40	40	15	54	25	17	24		98	11		23	
**8**			18	23	26	41	21	21	46	28	14	27		18	13			
**9**				20	27	38	30	17	66	23	22	27		27	10			
**10**				26	29	24	26	17	60	32	15	22		16	21			
**11**				41	28	38	29	16	36	27	16	24		22	11			
**12**				24	25	32	33	18	52	25	18	25		16	16			
**13**				22	26	29		17	48	27	14	20		62	14			
**14**				38					57	22	14			20	14			
**15**				44					71	28	14			37				
**16**				29					71		11			23				
**17**				20					42		20			27				
**18**				33							13			12				
**19**				17							19			18				
**20**											18							
**21**											16							
**22**											14							
**23**											17							
**24**											22							
**25**											14							
**26**											19							
**27**											20							

**28**											16							
**29**											13							
**Mean CV RMSSD in %**	17	20	16	27	31	35	30	18	54	26	17	22	24	31	13	35	24	19

	**Concussed**																	
**Subject ID**	3	5	9	10	11	13	14	17	18	19	20	21	22	24	27	29	30	31
**Nights**	**CV HR (in %)**																	
**1**	6	3	8	8	6	8	4	8	5	6	4	8	7	5	6	5	5	4
**2**	8	9	5	4	6	9	9	7	6	6	6	10	8	8	6	6	4	5
**3**	8	6		7	4	10	7	9	7	8	7	9	8	5	4	6	6	7
**4**	5	4		6	6	8	5	7	5	7	5	8	7	4	5	7	6	4
**5**		10		6	6	11	7	5	6	8	7	9	8	8		6	6	5
**6**		8		8	5	15		5	6	4	8	5	12	11		5		4
**7**		7		7		12		7	7	8	6	8	8	6		9		
**8**		5		6		12		7	7	4	7	9	7	7		6		
**9**		6		8		9		5	5	7	5	14	10	8		7		
**10**		5		7		11		8	5	8	8	9	6	7		7		
**11**		4		8				7	8	6	7	7	7	9		7		
**12**		7		9				10	6	8	6	8	7	5		7		
**13**		5		8					6	7	5	7	6	6		6		
**14**		5		6					7	7	8	7	10	3		7		
**15**				6					7		8	8	8	4				
**16**									6		7	7	8	6				
**17**									6		8	9	7	5				
**18**									7		5	9	9	8				
**19**											7	8	10	4				
**20**											7	8	7	5				

**21**											8	10	8	6				
**22**											6	7	7	10				
**23**											7	7	7	7				
**24**											6	7	8	9				
**25**											8	5	6	8				
**26**											6	8	13	7				
**27**											6	8	7	5				
**28**											10	9	6	5				
**29**											7	8	6	11				
**30**											5	8	8	3				
**31**											5	10	7	9				
**32**											6	9	7	12				
**33**											6	11	5	10				
**34**												10	7	7				
**35**												8	7					
**36**												10	8					
**37**												10	7					
**38**												9	7					
**39**												6	9					
**40**												12	8					
**41**												11	6					
**42**												11	6					
**43**												5	6					
**44**												6	9					
**45**												6	7					
**46**												6	8					
**47**												7	7					
**48**													6					
**49**													7					

Mean CV HR in %	7	6	7	7	5	10	6	7	6	7	7	8	8	7	5	7	6	5
	**Controls**																	
**Subject ID**	3.1	5.1	9.1	10.1	11.1	13.1	14.1	17.1	18.1	12.1	20.1	21.1	22.1	24.1	27.1	29.1	30.1	31.1
**Nights**	**CV HR (in %)**																	
**1**	7	5	5	5	4	9	8	7	6	7	5	5	5	12	4	10	4	3
**2**	8	5	6	5	5	7	5	7	7	8	3	4	6	6	5	8	8	5
**3**	5	5	6	6	13	16	6	7	11	5	3	4	4	4	7	10	4	9
**4**	7	3	5	7	9	12	7	7	9	6	6	4	3	4	5	14	3	5
**5**		4	6	6	9	9	8	5	12	5	6	7		8	4	6	3	3
**6**		4	4	6	9	17	9	6	10	7	5	5		8	8		3	6
**7**			7	10	6	13	12	6	7	7	4	4		9	4		3	
**8**			4	4	6	13	4	6	5	7	4	6		2	7			
**9**				5	5	9	8	7	10	6	4	6		15	5			
**10**				6	8	7	6	5	7	7	4	3		3	10			
**11**				7	5	10	6	7	8	7	5	4		4	7			
**12**				5	7	9	6	5	6	6	4	8		3	7			
**13**				6	6	7		6	7	9	5	5		6	4			
**14**				6					8	7	4			10	5			
**15**				6					8	7	2			9				
**16**				6					7		5			5				
**17**				6					7		6			6				
**18**				5							5			7				
**19**				5							5			8				
**20**											4							
**21**											4							
**22**											5							
**23**											5							
**24**											7							
**25**											5							

**26**											4							
**27**											5							
**28**											6							
**29**											5							
**Mean CV HR in %**	7	4	5	6	7	11	7	6	8	7	5	5	5	7	6	10	4	5
